# Streamlined process development using the Micro24 Bioreactor system

**DOI:** 10.1186/1753-6561-7-S6-P36

**Published:** 2013-12-04

**Authors:** Steve RC Warr, John PJ Betts, Shahina Ahmad, Katy V Newell, Gary B Finka

**Affiliations:** 1 Upstream Process Research, GlaxoSmithKline, Stevenage, SG1 2NY, UK

## Introduction

The Pall Micro24 Bioreactor system is one of several microbioreactor systems that have been commercialised in recent years in response to the demand to reduce costs and shorten process development time lines.

We have previously demonstrated that the Micro24 Bioreactor system can be integrated successfully into the later stages of cell line screening programmes and that the results correlate well with those from more conventional methods [1]. Further process development for these selected cell lines traditionally utilises bench top bioreactors to define appropriate process conditions giving the desired process outcomes although this approach can be time consuming and resource intensive. However the Micro24 Bioreactor system allows up to 24 different process conditions to be run concurrently thereby facilitating efficient process development.

This work describes the use of the Micro24 Bioreactor system to identify improved process conditions for different cell lines and their subsequent validation in bioreactors.

## Micro-24 bioreactor system (Pall)

This system comprises 24 bioreactors (7 ml working volume) each capable of independent temperature, dissolved oxygen and pH control. The main limitation of the system is the lack of automation meaning that any feed additions or sample removal must be made manually and similarly, for mammalian cultures, upwards pH control is achieved by the manual addition of NaHCO_3_.

Engineering characterisation studies carried out at UCL (data not shown) have shown how conditions within the individual Micro24 chambers compare with those in bioreactors and recent results also indicate that the selection of the Micro24 plate type is critical in ensuring good correlation with performance in traditional bioreactors.

Within the Micro24 Bioreactor system cell cultures are carried out in presterilised polycarbonate mammalian cell culture cassettes which are inoculated manually in a laminar flow cabinet before sealing with Type A single use closures and incubation under experimental conditions.

## Methods

Chemically defined medium and feeds were used throughout this work. Unless otherwise stated standard experimental conditions were used. (35ºC, pH 6.95, 30% Dissolved Oxygen (DO)). Viable cell numbers and viability were determined using a ViCell Cell Viability Analyser (Beckman Coulter) and antibody titres were determined using an Immage Immunochemistry System (Beckman Coulter).

## Process optimisation

Typical process relevant factors that can be tested in the Micro24 include feed regime, pH, DO and temperature. The effects of these types of factors are best tested using a Design of Experiments (DoE) approach to assess the effects not only of different factors but also of the interactions between them. Such data can then be used to build predictive models of process performance to specify the appropriate operating conditions in larger scale bioreactors. We have already developed and are using a similar approach for microbial dAb processes.

The data below shows examples of how we have used this system to identify improvements to platform processes for specific cell lines.

## Case Study 1 - process conditions

In this experiment the effects of changes to the platform process pH and DO set points on the performance of a mAb producing cell line were assessed in the Micro24 using a DoE approach with different operating conditions in each well.

This data demonstrated that although the dissolved oxygen level had little effect on viable cell numbers, titres and specific productivity, operating at a higher pH than the standard platform set point resulted in an increase in titre and in specific productivity. There was no significant interaction between the factors.

## Bioreactor validation (1) - 2 litre scale

The high pH process identified from the Micro24 was run in 2 litre bioreactors and compared to the standard platform process.

At the high pH set point cell numbers during the later stages of the process were slightly reduced compared to the control and as in the Micro24 higher titres were produced under higher pH conditions. However, as in the Micro24 the greatest effect of increased pH was on specific productivity which in the bioreactors was increased by approximately 35% compared to the control.

## Bioreactor validation (2) - 50 litre scale

**Figure 1:**
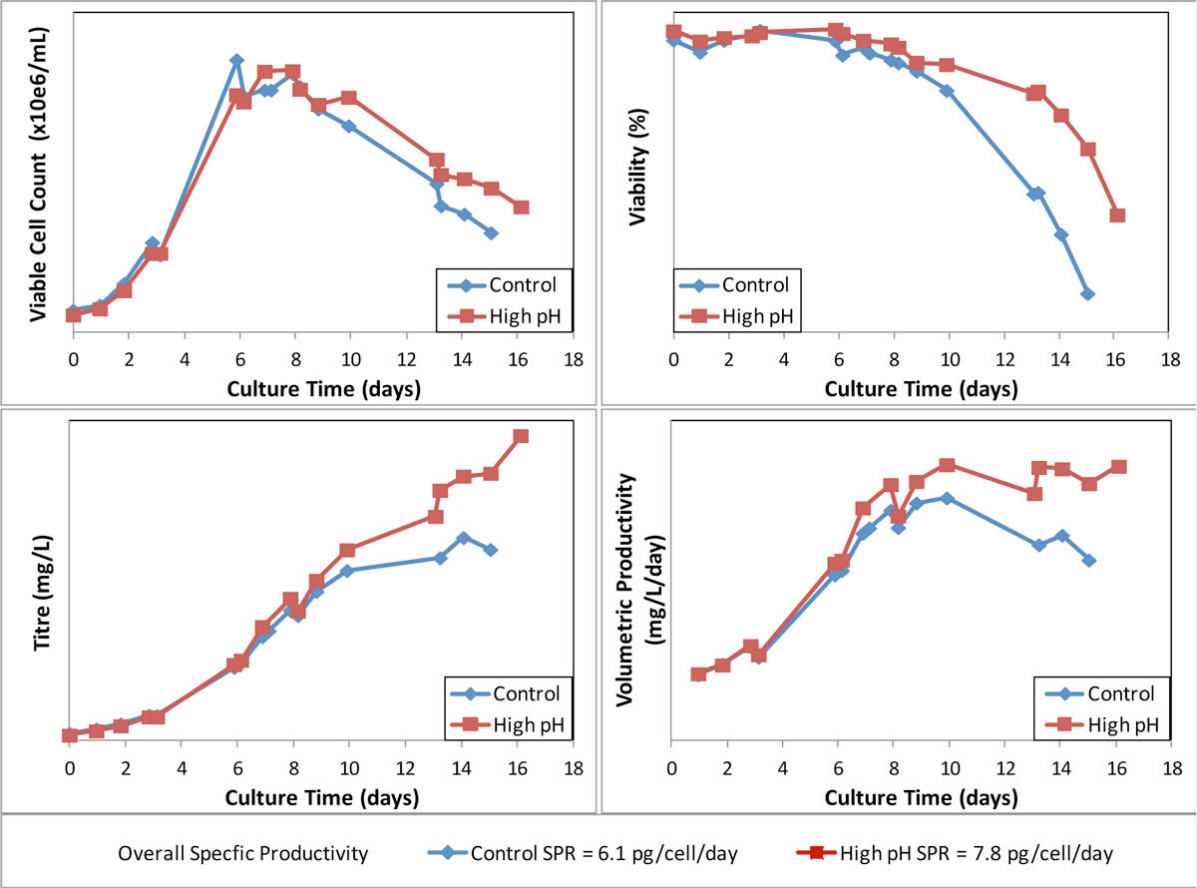
**Effect of pH on cell line performance in 50 litre bioreactors**.

Similar results were achieved at the 50 litre scale for a different cell line running in the same platform process but producing a different molecule (Figure 1). There was little effect on the cell numbers but the higher pH condition resulted in increased titre, culture duration, volumetric productivity and specific productivity.

## Case study 2 - feeding regime

The Micro24 can be used to investigate the effect of different feeding regimes on culture performance. We have already demonstrated that the effect of feed addition on culture performance in the Micro24 is similar to that in shake flasks [1]; the data below (Table 1) shows that for a chemically defined process multiple feed additions have a similar effect in 2 litre bioreactors to the Micro24. In both systems the addition of the feed results in significant increases in cell numbers and titre. Culture duration is increased and the overall specific activity is increased by 63% in the Micro24 and 79% in the bioreactors.

**Table 1:** Comparison of the effect of feed on cell line key performance parameters in Micro24 and 2 litre bioreactors

Effect of Feed on Key Performance Parameters in Micro 24 and 2 L Bioreactors					
		**Normalised VCC**	**Normalised Culture Duration**	**Normalised Peak Titre**	**Normalised SPR**

Micro 24	Unfed	100	100	100	100
	Fed	147	113	206	163
					
2 L Bioreactors	Unfed	100	100	100	100
	Fed	171	133	379	179

## Discussion

Our previous work has demonstrated how the Micro24 system can be used for mammalian cell line selection [1] and the data presented here extends the application of the Micro24 into mammalian process development. The parallel nature of the Micro24 enables process relevant factors to be tested in DoE experiments and these data show that improved process conditions such as increased pH and feed additions identified in the Micro24 can be used to achieve process improvements in bioreactors.

The validation of the Micro24 results in bioreactors suggests that the integration of this technology into mammalian process development could reduce significantly the numbers of bioreactors required to achieve process improvements which could result in reduced resource requirements and improved timelines.
